# Configurational Field Analysis: a new approach to global field dynamics

**DOI:** 10.3389/fsoc.2024.1496197

**Published:** 2024-12-12

**Authors:** Abbas Jong

**Affiliations:** ^1^Department of Social Sciences, Humboldt University of Berlin, Berlin, Germany; ^2^Department of History of Philosophy, Patrice Lumumba People’s Friendship University of Russia (RUDN University), Moscow, Russia

**Keywords:** social field, social configuration, global field theory, configurational field analysis, Pierre Bourdieu, indeterminacy

## Abstract

This paper advances the theory of Configurational Field Analysis (CFA) as a reconfiguration of Pierre Bourdieu’s field theory, aiming to address the limitations of Global Field Theory in analyzing the complexities of global and transnational phenomena. While the concept of the Global Field extended Bourdieu’s ideas to transnational and global arenas, it has been critiqued for its structural determinism, Eurocentrism, and its inability to fully capture the fluid, indeterminate, and contingent nature of global social dynamics. In response, this paper introduces social configurations as dynamic, relational constructs that emerge from specific historical and contextual conditions, rather than as fixed and universal structures. By integrating the concept of social configurations into field theory, CFA reconceptualizes social spaces as fluid and contested arenas where power, capital, and influence are continually negotiated. The paper proceeds by revisiting the foundations and critiques of Global Field Theory, followed by the introduction of social configurations and their theoretical advantages. Finally, it presents Configurational Field Analysis as a comprehensive framework, detailing its analytical steps and demonstrating its applicability to contemporary global issues. This framework not only addresses the methodological and analytical gaps in Global Field Theory but also offers a more adaptable and context-sensitive approach for understanding the complexities of global interactions.

## 1 Introduction

As the world becomes increasingly enmeshed in intricate networks of transnational flows, traditional social theories struggle to capture the fluid, indeterminate, and multifaceted realities of contemporary cosmopolitanization (Beck, 2006). The rigid, nation-state-centered paradigms that once explained social dynamics now face difficulties in accommodating the complexities of a world where power, capital, and cultural influence cross borders with unprecedented speed and unpredictability (Castells, 2010; Jong, 2023a). The concept of the Global Field, an extension of Pierre Bourdieu’s seminal field theory (Bourdieu, 1990; Bourdieu and Wacquant, 1992), aimed to address these global transformations by conceptualizing social spaces beyond national boundaries (Buchholz, 2017). However, as this theory has developed, so too have critiques of its ability to fully capture the complexities of transnational social fields and the fluidity of global power dynamics (Go, 2008; Jong, 2024).

Despite its promises, Global Field Theory has faced criticism for its structural determinism (Jenkins, 2002; King, 2020), reproductionism (Beck, 2016), Eurocentrism (Go, 2013), and its inability to account for the fluid, indeterminate nature of global and cosmopolitanized social phenomena (Jong, 2024). Critics argue that while its conceptual apparatus is sophisticated, it often reifies existing structures and overlooks the dynamic interactions that characterize global fields (Beck, 2006). Additionally, the theory’s abstraction limits its empirical utility, making it challenging to operationalize and apply to the increasingly complex and interconnected realities of the global landscape (Jong, 2024; Levitt and Khagram, 2007). These critiques suggest that while the concept of the Global Field offers valuable insights into transnational dynamics, it requires further refinement to fully capture the nuanced and evolving nature of global social phenomena.

This paper argues that overcoming the limitations of Bourdieu’s field theory and Global Field Theory necessitates a fundamental reconfiguration, grounded in a novel ontological and epistemological framework. By introducing the concept of “social configurations,” this study proposes an alternative approach that more effectively captures the fluidity, indeterminacy, and contingency inherent in global and transnational phenomena. Unlike the traditional unit of fields, social configurations—here serving not as an analytical unit but as a logic for constructing social units—are dynamic, relational constructs that emerge from specific historical and contextual conditions rather than adhering to fixed, universal principles. By integrating these configurations into field theory, this paper advances Configurational Field Analysis (CFA), a comprehensive approach that transcends the constraints of both traditional and global field theories.

Configurational Field Analysis reconceptualizes fields as dynamic, contextually embedded spaces where power, capital, and influence are continuously contested and reconfigured. This approach not only addresses the methodological and analytical gaps present in Global Field Theory but also enhances our capacity to explain and understand the fluid and evolving nature of global and transnational interactions. Through CFA, this paper seeks to advance a more nuanced and adaptable framework for analyzing global dynamics—one that is both theoretically rigorous and empirically grounded.

The structure of this paper proceeds as follows: First, it introduces and revisits the foundations and critiques of Global Field Theory, evaluating its contributions and limitations in analyzing global social phenomena. Second, it presents the idea of social configuration, delineating its key characteristics and theoretical advantages. Finally, the paper introduces Configurational Field Analysis as a comprehensive framework, outlining its analytical steps and demonstrating its applicability to contemporary global issues. By reconstructing the concepts of field, capital, and habitus within the more fluid and dynamic framework of configuration, this paper aims to refine our understanding of global phenomena in an increasingly complex, uncertain, and interconnected world.

## 2 Global field theory

The concept of “Global Field” extends and complicates Pierre Bourdieu’s field theory by shifting its focus from national boundaries to transnational arenas, where social, economic, political, and cultural dynamics unfold (Go, 2008). This theoretical extension necessitates a reconsideration of how actors compete for various forms of capital—not just within nation-states but across a broader global landscape, where interactions are increasingly mediated by global institutions, transnational networks, and cross-border flows of capital and ideas (Levitt and Schiller, 2004). A global field, therefore, is not simply an expanded version of national fields; it represents a distinct set of social spaces where power, hierarchy, and competitive struggles are structured by global logics rather than localized or national ones (Beck, 2006). This shift underscores the need for sociological theories to account for the unique dynamics of globalization and cosmopolitanization.

Pierre Bourdieu’s original field theory conceptualizes social spaces where agents and institutions compete for various forms of capital—economic, cultural, social, and symbolic—each field governed by its own internal logic and rules (Bourdieu, 1984, 1990; Bourdieu and Wacquant, 1992). While the theory was primarily developed within the context of nation-states, scholars have sought to explore its applicability in a globalized world. This exploration reveals significant complexities that necessitate a reconceptualization of traditional notions of fields. As emphasized, global fields are not merely scaled-up versions of national fields; rather they represent trans-national arenas shaped by distinct logics, structures, and power relations. Expanding Bourdieu’s theory to the global level requires acknowledging how global dynamics reconfigure competition for various forms of capital and create new hierarchies that transcend national boundaries. This concern has been the primary focus of many scholars who have attempted to formulate the concept of the global field.

One crucial extension of Bourdieu’s field theory is the recognition of the core-periphery structure that defines many global fields, as highlighted by Heilbron (2013). Heilbron argues that in fields such as the global social sciences, a small number of core actors—primarily from North America and Europe—dominate knowledge production and dissemination, while semi-peripheral and peripheral regions possess limited influence and access to resources. This core-periphery dynamic is not merely a reflection of national power differentials; rather, it is institutionalized within the global field itself, reinforcing the uneven distribution of symbolic and material capital on a global scale.

Go (2008) extends this discussion by linking the structure of global fields to imperial forms. He argues that global fields are shaped not only by contemporary transnational processes but also by the historical legacies of empire. Go illustrates how global political and economic fields were historically structured by imperial competition for dominance. These imperial forms, rooted in European colonialism, have left a lasting imprint on today’s global fields, creating a layered and hierarchical structure that reflects the power relations of past empires. Therefore, Go underscores the importance of understanding global fields not merely as products of modern globalization but also as continuations of historical imperial forms that still shape global hierarchies of power.

Buchholz (2017) builds on this discourse by introducing the concept of “vertical autonomy” to capture the partial independence that global fields maintain from national fields. Vertical autonomy refers to the ability of global fields to develop their own evaluative criteria and forms of capital that transcend national boundaries, while still remaining connected to them. In her work, Buchholz identifies three essential components of global fields: global institutions, transnational discourses, and global evaluation mechanisms. Global institutions, such as UNESCO or the World Trade Organization, play a pivotal role in shaping global norms and standards. Transnational discourses, circulating across borders, create shared narratives and frameworks that influence how global actors perceive and engage with the world. Finally, global evaluation mechanisms, such as international rankings or awards, offer ways to assess and legitimize the distribution of capital on a global scale. Together, these three aspects reinforce the autonomy of global fields, allowing them to establish hierarchies of power and influence that extend beyond national contexts. For instance, in the global art field, these mechanisms shape the recognition of artists not only within their national traditions but also on the global stage.

Steinmetz (2016) adds a crucial historical dimension to this discussion by examining the formation of global fields on the scale of empires. He argues that the structures of global fields are profoundly influenced by the legacies of colonialism and imperialism, which have left lasting imprints on the logics and hierarchies within these fields. Consequently, the global field is a product of historical struggles and uneven power relations that continue to shape its contours. This historical perspective underscores that global fields are not static entities; rather, they are continually evolving in response to shifts in global power dynamics.

Go and Krause (2016) further develop this argument by illustrating how global fields are constituted through the interplay of various actors and institutions across multiple scales. They contend that global fields are relational spaces where power is contested and negotiated through interactions between local, national, and transnational actors. This multi-scalar approach challenges the methodological nationalism that often pervades social theory, proposing that a more nuanced understanding of global fields requires acknowledging the interconnectedness of these different levels.

Adding to this discussion, Wimmer (2021) provides valuable insights into how global fields are shaped by cultural diffusion and institutional travel. Although Wimmer only briefly addresses global fields and tries to go beyond it, his work emphasizes the significance of understanding how cultural forms and institutions migrate and transform across borders, leading to the restructuring of global fields. Wimmer’s framework underscores the dynamic and contingent nature of global fields, where transnational diffusion plays a critical role in reshaping social spaces, creating new forms of capital, and altering competitive dynamics.

Lastly, Lim (2021) examines the emergence and operation of global fields in the context of transnational corporations and institutional regulation. Lim argues that global fields are characterized by the interaction between institutional actors and global governance structures. In the case of transnational corporations, these actors navigate global regulatory frameworks that shape the distribution of capital and power within the global economic field. Lim’s work offers a concrete example of how global fields are structured not only by symbolic and cultural capital but also by institutional rules and economic power, further enriching our understanding of global field theory.

The complex interplay between power hierarchies, historical legacies, vertical autonomy, and regulatory frameworks, as highlighted in the existing studies on global field, defines the core components and dynamics of global fields. These elements work together to shape the distribution of capital and influence within global arenas, reinforcing the inequalities embedded in these transnational spaces. Understanding global fields requires attention to the fluid and contingent nature of these dynamics, where actors continually compete for power, capital, and influence within ever-shifting global contexts.

### 2.1 Methodological and analytical implications

The studies on global fields accompanied by a critical rethinking of sociological methodologies, particularly by challenging the constraints of methodological nationalism and other methodological biases and forms of centrism. As globalization increasingly blurs the boundaries between the local, the national and the global, traditional approaches that privilege the nation-state and national actors as the primary unit of analysis prove insufficient for capturing the complexities of global interactions (Jong, 2022). Moving beyond these limitations requires adopting multi-scalar methodologies that recognize the intricate linkages between local, national, transnational and global dynamics. This approach allows scholars to analyze global fields not as isolated or purely global entities but as deeply interconnected with other scales of social life. Go and Krause (2016) highlight that field theory offers a valuable tool for overcoming the limitations of existing approaches, such as world-systems theory, by focusing on the relational dynamics that operate within and across fields.

Incorporating field theory into global studies necessitates contextual and historically specific analysis, as Steinmetz (2016) argues. Steinmetz’s exploration of global fields at the scale of empires underscores the importance of historical processes in shaping the structures and logics of global fields. These fields do not emerge in a vacuum; rather, they result from long-standing power struggles and historical contingencies that continue to influence their development. Consequently, research on global fields must be grounded in empirical studies that account for the historical trajectories and power relations that shape these fields. This approach challenges any *a priori* assumptions that global fields are stable or coherent, instead revealing their dynamic and contested nature.

The methodological shift toward multi-scalar and relational analysis significantly impacts our understanding of the diffusion of culture and institutions across global fields. Andreas Wimmer (2016) suggests that understanding the spread of cultural products and institutional forms requires analyzing how global and local actors negotiate these diffusions within specific contexts. By moving beyond simplistic diffusion models, scholars can better capture how global fields are continually reshaped by interactions between different scales and actors. This perspective aligns with the need for more flexible and adaptive research methods that account for the contingent and evolving nature of global fields, as well as the multiple forces driving their transformation.

Furthermore, Lim’s (2021) analysis of institutional emergence within global fields underscores the importance of understanding how global governance structures evolve. Lim’s work suggests that methodological approaches must be attuned to the processes by which global institutions and norms are constructed and contested within global fields. This involves examining the complex interactions between regulatory frameworks, global actors, and local contexts. By adopting methods that are sensitive to these dynamics, researchers can uncover the underlying mechanisms driving the evolution of global governance within global fields, thereby offering deeper insights into how power operates on a global scale.

### 2.2 Efficiency and specialty of global field

The theory of global fields offers a sophisticated and comprehensive framework that transcends the limitations of traditional theories in understanding global interactions. Traditional approaches, such as Realist International Relations (RIR) and Hegemony/World Systems (HWS) theory, are often criticized for their state-centric focus and deterministic views on global economic hierarchies, respectively. RIR, as proposed by Waltz (1979), views the international system primarily as a competitive arena for self-interested states, emphasizing power dynamics without fully accounting for the complex interdependencies and shared norms that shape state behavior in today’s interconnected world (Wendt, 1999). Similarly, HWS theory, influenced by Wallerstein (1974), focuses on economic hierarchies and core-periphery relations but often overlooks the cultural and normative dimensions that influence economic systems and the agency of diverse actors in driving global change (Chase-Dunn and Grimes, 1995). The theory of global fields addresses these critical gaps by integrating material capabilities, cultural norms, and social structures into a cohesive analytical model that more accurately reflects the complexities of global interactions (Buchholz, 2017).

Global fields theory also critiques the limitations of contemporary theories such as Complex Interdependence, Network Theory in International Relations, and World Society Theory. Complex Interdependence, as articulated by Keohane and Nye (2001), challenges the state-centric focus of RIR by emphasizing the role of non-state actors, such as multinational corporations and NGOs, in shaping global governance. However, this approach may still fall short in capturing the deep-seated cultural and normative forces that global fields theory emphasizes. Similarly, Network Theory, which focuses on connectivity, information flows, and relational power structures (Hafner-Burton et al., 2009), aligns with global fields theory by recognizing the significance of networks in shaping global interactions. Yet, global fields theory extends this understanding by emphasizing how these networks are embedded within broader cultural and social structures that influence both state and non-state actors’ behaviors (Go and Krause, 2016).

Moreover, World Society Theory, as proposed by Meyer et al. (1997), suggests that global cultural norms, often Western in origin, serve as templates for legitimacy and governance worldwide. However, this theory is critiqued for underestimating the local resistance and reinterpretation of these norms—a gap that global fields theory effectively addresses by incorporating the agency of diverse actors in shaping global cultural landscapes (Robertson, 1992). Global fields theory offers a nuanced framework for understanding cultural globalization as a dynamic and negotiated process, considering how global norms are constructed and contested by various actors, including states, NGOs, and transnational networks. Additionally, global fields theory can be extended to address paradigms such as the transnational capitalist class (Sklair, 2001) and environmental governance (Newell, 2000). It examines how global economic fields are structured through both economic power and cultural hegemony, revealing the interplay between transnational capital and local adaptations. In environmental politics, global fields theory provides insights into how environmental norms are constructed and contested, emphasizing the dynamic interplay between global and local practices.

The study of international organizational models illuminates how institutional practices and policies are transmitted and adapted across different regions, with a focus on the processes of institutional change. Traditional models of policy transmission often emphasize linear paths of adoption, assuming that best practices are uniformly transferable across contexts. However, this approach frequently overlooks the transformative changes and contextual adaptations that occur as these practices are implemented in diverse settings (Strang and Soule, 1998). Global fields theory offers a critical perspective by incorporating insights from network theory and social capital, emphasizing that the transmission and adaptation of organizational models are shaped by relational ties and power structures within global fields. These relational dynamics suggest that global organizational practices are not simply transferred but are actively interpreted and transformed within different social and institutional landscapes. This perspective provides a more comprehensive framework for analyzing the complexities of global interactions, revealing the diverse pathways through which organizational models evolve and adapt in the global arena. By integrating these insights, global fields theory facilitates a deeper understanding of how institutional practices are negotiated and reshaped across different contexts, leading to varied outcomes (Powell and DiMaggio, 1991).

In comparison to traditional and contemporary theories, global fields theory offers a more comprehensive and integrative approach by emphasizing the dynamic interplay between material, cultural, and structural dimensions of global interactions. It acknowledges the agency of a wide range of actors, from states to non-state entities, in shaping global fields, and underscores the importance of historical and contextual specificity in understanding global dynamics (Steinmetz, 2016). This approach enables a more nuanced analysis of global interactions, revealing the complex interdependencies, contested cultural norms, and evolving power relations that define the global arena. As such, global fields theory not only critiques the limitations of existing theories but also provides a robust framework for understanding the complexities of globalization in the 21st century (Go, 2008).

### 2.3 Critiques of Bourdieu’s global and social fields

While Bourdieu’s theory of fields offers a valuable framework for understanding social dynamics, its application to global contexts has faced significant critiques. These criticisms, which include concerns about theoretical rigidity and Eurocentrism, question the comprehensiveness of global fields theory when addressing the complexities of globalization. Such critiques highlight the need for further refinement and adaptation of the theory to more fully account for the fluid, diverse, and interconnected nature of global social phenomena.

One substantial critique is that global fields theory oversimplifies cultural diffusion by assuming structured and predictable interactions within fields. In reality, cultural practices often mutate as they cross borders, defying the coherence that global fields theory seeks to impose (Appadurai, 1996; Wimmer, 2016, 2021). Additionally, the theory’s emphasis on structured fields and relational dynamics may lead to structural determinism, where actors’ roles are seen as overly constrained by the structures of the field. Critics argue that this focus limits the recognition of individual and collective agency in shaping and transforming fields, particularly in global contexts where non-state actors, such as multinational corporations and transnational networks, play crucial roles (Giddens, 1984).

Global fields theory has also been critiqued for its abstraction and overgeneralization, which can undermine its empirical utility. While the theory’s emphasis on relational dynamics and power structures within global fields is conceptually rich, it often lacks the empirical specificity needed to effectively operationalize these ideas in sociological research (Steinmetz, 2016). Critics argue that although the theory provides a broad framework for understanding global interactions, it can be difficult to delineate the concrete boundaries of global fields, as well as the specific actors and processes involved. This abstraction can limit the theory’s practical utility, making it challenging to apply in empirical studies that require clear definitions and measurable variables.

Additionally, scholars like Fligstein and McAdam (2012) have critiqued global fields theory for attempting to create a unified model that applies across different domains—economic, political, and cultural—potentially oversimplifying the unique logics that operate within each of these spheres. The result is a theory that may be too broad to capture the specific dynamics at play in different areas of global life, such as the distinct mechanisms governing global financial markets versus those influencing global cultural exchanges. This overgeneralization risks diluting the explanatory power of global fields theory, making it less effective at addressing the nuances of particular global phenomena.

Bourdieu’s original theory of social fields also faces significant challenges. Critics argue that Bourdieu’s emphasis on structure limits the role of individual agency, particularly in global contexts where non-state actors like multinational corporations reshape global norms (Sklair, 2001; Lim, 2021). Additionally, Bourdieu’s framework struggles to account for the rapid social changes characteristic of globalization, raising questions about its relevance in fast-evolving global fields (Friedman, 2004). Similarly, Ulrich Beck critiques Bourdieu’s concept of social fields as embodying reproductionalism in social theory, which, according to Beck, lacks the capacity to understand and conceptualize social phenomena within the accelerating and complex interwoven networks and transformations of today’s world. As a result, Bourdieu’s approach may appear conservative, particularly in addressing global phenomena defined by their inherent indeterminacy, divergences, crises and fluidity (Beck, 2016). Furthermore, the Eurocentric bias in Bourdieu’s concepts—developed in the context of Western, primarily modern societies—raises concerns about their applicability to non-Western and non-modern contexts, where different forms of capital may prevail (Swartz, 1997).

Further criticisms suggest that global fields theory risks reifying the very structures it seeks to analyze, treating fields as stable entities rather than as fluid and evolving spaces. This reification limits the theory’s ability to fully capture the dynamic nature of global interactions (Buchholz, 2017; Latour, 2005). From an epistemological perspective, it can be argued that social fields perpetually suspend the indeterminacy of phenomena that are in a constant state of becoming, thereby sacrificing their potentiality and historicity. This represents another aspect of hidden structuralism within the theory of social fields. An important critique of Eurocentrism surrounding the idea of the social field questions its validity and core components, which are closely aligned with liberal democracies, Western rational actors, and an inherent colonial nature. This critique underscores the need for a postcolonial suspension, marking it as a significant point of contention regarding this concept (Go, 2013). By challenging Eurocentric biases, a revision idea of social field can facilitate culturally sensitive analyses that incorporate non-Western perspectives, expanding the applicability of field theory to diverse global contexts (Beck, 2000; Quijano, 2007; Go, 2013). Additionally, global fields theory may overemphasize relational dynamics at the expense of broader structural forces, such as global capitalism or state power. While global fields theory excels at analyzing interactions between actors within a field, it may underplay the significance of these macro-structural forces that operate beyond the immediate relational dynamics of the field. Lastly, the focus on competition within fields may inadvertently naturalize competitive logics, reinforcing global inequalities rather than exploring alternative frameworks that prioritize cooperation and solidarity (Harvey, 2005). These critiques underscore the necessity of a fundamental revision of the idea of the social field in general, and the global field in particular, in relation to new and rapidly evolving global contexts. This reassessment aims to retain the unique advantages of the concept while enhancing its analytical capacity.

## 3 The idea of social configurations

Social configurations can provide an alternative conceptual framework to the traditional notion of fields, particularly in relation to the increasingly interwoven and indeterminate global context mentioned earlier, and in addressing and overcoming the prevailing issues within the ideas of the social and global fields. Unlike Bourdieu’s fields, which are often viewed as relatively stable, homogenous, bounded, and structurally coherent entities, social configurations are conceived as dynamic, fluid, indeterminate, heterogeneous, and contingent. Social configurations, unlike existing analytical units—typically predefined and standardized—function primarily as a logic for constructing the social or social units within a context of perpetual becoming and indeterminacy, embedded in a web of interwoven and reciprocal relations. They reflect a relational and post-foundational ontology, where social reality is understood as a network of interconnected and evolving relationships around constructed categories, rather than as discrete and isolated units. Social configurations are in a constant process of formation, temporally actualized within a constellation of relations at specific moments and based on incomplete and partial foundations (Jong, 2023b, 2024; Emirbayer, 1997).

In defining social configurations, several key characteristics emerge.

Relationality: Relationality constitutes the fundamental principle of social configurations, emphasizing that social entities derive their identity and significance not from inherent or essential traits but from their positions within broader relational networks. This perspective shifts analysis from static categorizations to the dynamic processes through which social realities are constructed and continuously reconfigured (Jong, 2023b, 2024; White, 1992). Configurations emerge through the interplay of specific actors within temporally and spatially defined contexts, transcending established scales, categories, and relationships. While overlaps may occur, configurations manifest as provisional units, contingent on their stability, structuration, and regularity at a given moment. Crucially, all these parameters are inherently relational, subject to variability and redefinition based on the dynamics of interaction.

Contingency: Social configurations are fundamentally characterized by their contingency, meaning they emerge and evolve within specific historical, cultural, and situational contexts. This contingency is grounded in the philosophical framework of post-foundationalism (Jong, 2023b), which rejects the rigidity of foundationalist approaches and the nihilistic tendencies of anti-foundationalism. Post-foundationalism offers a middle ground, conceptualizing social configurations as provisional constructions shaped by the interplay of (non-)historical relationships and contextual forces at a given moment. Central to this perspective is the dual recognition of both the incompleteness of their foundational bases and the openness to alternative configurations. This duality underscores the dynamic nature of social configurations, wherein their grounding processes are never final but always contingent, reflecting specific conditions while simultaneously allowing for the indeterminacy of unrealized possibilities (Butler, 1992; Sewell, 1992). Such a perspective contrasts sharply with deterministic frameworks that view social structures as fixed or enduring. Instead, the contingency of configurations emphasizes their capacity for adaptability and transformation, contingent upon the fluidity of the contexts in which they arise. This adaptability is critical for understanding the variability of social configurations across different contexts, highlighting their capacity to respond dynamically to evolving historical and situational forces.

Fluidity and Indeterminacy: The inherent fluidity and indeterminacy of social configurations mark a significant departure from the fixed and bounded constructs characteristic of traditional field theories. Unlike these traditional approaches, which often presuppose clear boundaries and stable internal logics, social configurations are inherently dynamic, open to reconfiguration, and shaped by the interplay of diverse and evolving forces. Pierre Bourdieu’s field theory, while offering a robust relational framework, operates within an essentialist paradigm that assumes fields to be discrete and transhistorical entities with universal characteristics (Jong, 2024; Beck, 2016). Such a perspective risks overlooking the heterogeneity and continuous evolution of social phenomena, particularly in the context of global and transnational interactions. In contrast, social configurations are distinguished by their capacity to integrate novel elements and restructure relational dynamics in response to changing contexts. This adaptability underscores their relevance in analyzing complex global realities, where traditional scales and boundaries are increasingly blurred. By accommodating the fluid and contingent nature of social phenomena, configurations provide a more nuanced framework for understanding the intricate and interconnected nature of transnational and cosmopolitan processes.

Historicity: Social configurations are fundamentally embedded in historical processes, with their emergence, persistence, and transformation shaped by specific historical moments and contextual conditions (Jong, 2023b; Foucault, 1977; Hobsbawm, 1994). This historicity applies not only to the observable manifestations of configurations but also to their internal structures, which evolve dynamically in response to temporal and contextual shifts. The contingent nature of configurations encompasses a broad spectrum of elements, including structural arrangements, agency dynamics, power relations, and resource distributions. Each of these elements is temporally situated, reflecting the historical specificity of the conditions under which the configurations actualized. This historical grounding challenges notions of universality and timelessness in social analysis, emphasizing the variability and contextuality that define configurations. Drawing on Wittgenstein’s concept of “family resemblance,” this perspective further underscores the rejection of fixed laws or relationships, advocating instead for an approach that recognizes the historically contingent and relational nature of configurations. By highlighting their historicity, this framework provides a robust lens for examining the temporal and contextual nuances that underpin the evolution of social configurations.

Nesting and Overlap: Social configurations, as mentioned, are not isolated entities; they often overlap and nest within one another. This nesting reflects the complex, multi-scalar nature of social reality, where local, national, and global configurations interact and influence each other. The concept of nesting is particularly important in the context of cosmopolitanization, where different scales of social life are increasingly interconnected, and social configurations at one level can have significant implications for those at other levels (Jong, 2024; Castells, 1996; Brenner, 2004; Beck, 2006).

### 3.1 Ontological and epistemological foundations

The conceptual shift from static fields to dynamic social configurations requires a recalibration of the theoretical underpinnings that frame social analysis. This section outlines the distinct ontological and epistemological foundations of social configurations, illustrating their role in addressing limitations inherent in Bourdieu’s field theory and advancing the analytical framework to account for the fluid and interconnected nature of global phenomena.

#### 3.1.1 Ontological foundations

The ontology of social configurations is rooted in relational and processual logics, challenging the structural determinism and boundedness that characterize traditional field theory. Rather than treating social entities as pre-existing or self-contained units, social configurations are conceived as emergent constructs that form and transform through relational dynamics in specific historical and contextual conditions (Jong, 2024; Latour, 2005). Unlike the fixed hierarchies of fields, social configurations operate through open and indeterminate boundaries that allow for fluid interactions across scales and categories. These configurations do not merely occupy predefined spaces; they continuously reconstruct their positionality through reciprocal influences. This openness reflects the realities of a globalized world where actors, forces, and networks intersect in ways that cannot be captured by rigid or static frameworks (Sassen, 2006). The relational ontology of configurations foregrounds temporality and historicity as core dimensions. Configurations are not abstracted from their historical trajectories or contextual contingencies; instead, they are dynamically situated within evolving socio-political and cultural landscapes. This ontological reorientation captures the multiplicity of relational processes that simultaneously define and destabilize the boundaries, identities, and logics of social configurations.

#### 3.1.2 Epistemological foundations

Epistemologically, social configurations advance a post-foundationalist approach that dismantles the universality and rigidity of traditional categories. In place of predetermined analytical frames, social configurations emphasize context-specific, historically grounded, and contingent analyses that engage directly with the complexities of global phenomena (Jong, 2023b, 2024; Foucault, 1972). This approach does not merely critique the foundationalist and anti-foundationalist paradigms underpinning much of classical social theory, including Bourdieu’s field theory; it transcends them by reconfiguring the analytic lens toward the indeterminacy and multiplicity of social interactions. Social configurations resist essentializing social phenomena, focusing instead on the fluid interplay of relations and the contingent conditions under which configurations emerge, dissolve, or transform. Importantly, the epistemology of social configurations allows for a critical engagement with unrealized possibilities—those configurations that remain unactualized within specific contexts. By examining the conditions that enable or constrain the emergence of alternative configurations, this framework broadens the scope of analysis, integrating the latent indeterminacies that underpin global social life. This flexibility is essential for capturing the contested and dynamic nature of contemporary global phenomena (Castells, 2010).

While Bourdieu’s field theory offers a valuable relational framework, its conceptual and methodological limits become evident in transnational contexts. The theory’s reliance on rigid field boundaries and its inability to fully address fluidity, contingency, and nested interactions within global phenomena restrict its applicability. Refinements introduced by global field theory have only partially mitigated these issues, as they largely retain the ontological and epistemological assumptions of the original framework (Beck, 2006; Jong, 2024). Social configurations address these limitations by reimagining fields as fluid and relational constructs, whose boundaries and logics are continuously redefined by their historical, cultural, and situational contexts. This shift from fixed fields to dynamic configurations enhances the framework’s capacity to account for the complexity of global interactions, particularly where actors and forces transcend traditional scales and categories.

Building on these ontological and epistemological reconceptualizations, this paper proposes Configurational Field Analysis (CFA) as a unified framework that synthesizes the relational principles of social configurations with the analytical strengths of field theory. CFA provides a methodological pathway for analyzing global phenomena in a way that preserves the rigor of field analysis while addressing its limitations. By integrating the open-ended and historically grounded logic of social configurations, CFA advances a dynamic and adaptable approach to understanding global social life. This integration facilitates a deeper engagement with the evolving nature of power, agency, and structure in contemporary contexts, bridging the gap between traditional sociological paradigms and the emergent realities of an interconnected world.

## 4 Configurational Field Analysis—an integrated approach

This section will analytically detail the integration of Pierre Bourdieu’s social field theory with the concept of social configurations. Through this process, the core concepts, categories, and relationships within social field theory will be redefined and reconceptualized through the lens of social configurations, resulting in what this research refers to as “Configurational Field Analysis.” This integration will not only reconfigure Bourdieu’s original concepts—Field, Capital, and Habitus—but will also adapt them to account for the dynamic, fluid, and contingent nature of social configurations. Gorski’s schematic model of Bourdieu’s social field serves as the foundation for this integration, as it synthesizes the essential parameters of Bourdieu’s field theory while expanding it into a more complex and historically grounded analysis (Gorski, 2013). Gorski’s model enables the integration of both the objective and subjective dimensions of fields, offering a relational and context-sensitive approach that is well-suited to examining the continuous transformation of social structures. The forthcoming discussion is structured in two parts. First, the general idea of Configurational Field Analysis will be introduced, outlining its dynamics, characteristics, and analytical stages. Second, an in-depth analysis will be conducted to demonstrate how Bourdieu’s concepts of fields, capital, and habitus are reconstructed through their integration with social configurations. This process will highlight the distinctive features of this new analytical framework and its potential to offer a deeper understanding of contemporary social realities.

### 4.1 Definition and dynamics

#### 4.1.1 Dynamic spaces

Configurational Field Analysis (CFA) conceptualizes fields as dynamic social spaces where actors, elements, and resources interact within specific historical and relational contexts. Unlike static interpretations of fields as fixed arenas, CFA emphasizes the fluidity and indeterminacy inherent in social configurations. These fields are characterized by evolving positions, power relations, and distributions of various forms of capital, creating a structured yet adaptable arena for social action. This dynamism allows fields to continuously respond to and incorporate new influences, making them highly responsive to shifting social, cultural, and historical conditions.

The concept of indeterminacy and fluidity is central to CFA. It challenges the more static models of social fields by emphasizing how fields are constantly reshaped through interactions among actors and the distribution of capital. The fluid nature of these fields permits creative agency and innovation, as actors navigate and redefine their positions within the ever-changing landscape of social configurations. This dynamic interaction leads to the continuous transformation of the field itself, making social configurations inherently contingent.

#### 4.1.2 Relational structure

Fields within CFA are defined by the complex interconnections among actors, forces, and elements. These relational structures are not simply networks of relationships; they form the very fabric that shapes interactions and outcomes within the field. The intricate web of relationships between different actors and forms of capital underscores the importance of understanding how these connections influence power dynamics and the overall configuration of the field. These fields function as interactive networks where positions and power are constantly negotiated. Actors leverage various forms of capital—economic, cultural, social, and symbolic—to influence and reshape the field’s configuration. The continuous negotiation of these positions within the relational network underscores the dynamic and non-linear nature of power in configurational fields. Consequently, understanding the relational structure of a field is crucial for analyzing how power is distributed and how fields evolve over time.

#### 4.1.3 Context-dependence

Configurational fields are deeply embedded in historical and cultural contexts. Layers of past events and socio-cultural dynamics significantly shape current structures and interactions within the field. This context-dependence underscores the importance of considering the temporal and spatial dimensions that influence social configurations. By situating fields within their specific historical and cultural frameworks, CFA enables a more nuanced understanding of how fields are shaped by broader societal changes. The adaptive capacity of configurational fields is another key characteristic. Fields are not static; they evolve in response to changing environmental and contextual factors. This adaptability is crucial for understanding how fields transform over time and incorporate new elements, ideas, and practices. The ability to adapt ensures that fields remain relevant and responsive to ongoing changes in the broader social landscape.

#### 4.1.4 Heterogeneity and openness

Configurational fields are characterized by heterogeneity and openness. They encompass a diverse range of elements and actors, fostering flexibility and openness to new configurations and influences. This diversity promotes innovation and the emergence of new configurations within fields, as different actors contribute varying forms of capital and strategies. The fluid boundaries of these fields further enhance their potential for innovation, enabling interactions and exchanges with neighboring fields, which can drive the evolution and transformation of the field itself. The fluid boundaries of configurational fields underscore their openness to external influences. These boundaries are not rigid but permeable, allowing for the incorporation of new ideas and practices from outside the field. This openness is crucial for understanding how fields evolve in response to external pressures and how they interact with other fields to create new configurations and relationships.

#### 4.1.5 Indeterminacy and contingency

Configurational fields are characterized by non-linear dynamics and contingent structures. The boundaries of fields, the types of capital and their rates of changes, the nature of their structures, the dominant categories in the fields and their orders, power relations within them, rules of game and other such aspects are all understood retrospectively within fields. These are seen as temporary and entirely based on unfinished foundations (continuously evolving), shaped by a specific constellation of relationships and forces at a given time and place. Small changes in relationships or conditions within a field can lead to significant shifts in its configuration, reflecting the indeterminacy central to CFA. This indeterminacy allows for the possibility of unexpected outcomes and innovations, as fields are not governed by rigid rules but are continually reshaped by the interactions within them.

### 4.2 Analytical steps in configurational fields analysis

The analytical process of Configurational Fields Analysis (CFA) is designed to systematically explore and map the dynamics of fields, their interactions, and the broader social configurations they inhabit. This process involves several interconnected steps that provide a detailed understanding of the structure, relationships, and transformations within fields. These steps are grounded in both empirical research and theoretical considerations, offering a robust methodological approach for analyzing social configurations.

#### 4.2.1 Step 1: identifying configurations

The first step in CFA is identifying configurations within a specific context. Unlike traditional field analysis, which may focus on predefined categories or structures, CFA requires a more open-ended and flexible approach. Configurations in CFA emerge based on the needs, desires, and interests of actors within specific social contexts. These initial interests spark social actions among actors, leading to the social interactions and formation of configurational fields. The process begins by identifying these needs and interests, understanding how actors perceive them, and mapping the types of interactions that arise from these perceptions. This step also involves examining the conditions of possibility that enable these needs or interests to emerge—conditions rooted in both historical and structural contexts. Simultaneously, the conditions of impossibility for other potential configurations must be analyzed, recognizing that not all possibilities are realized within a given social landscape.

For example, consider the global fintech configuration. This field has emerged from the convergence of technology and finance, driven by actors such as tech companies, financial institutions, regulators, and consumers. The configuration is shaped by a shared interest in developing innovative financial technologies, including mobile banking, digital payments, and cryptocurrency. Identifying this configuration requires mapping the interactions among these diverse actors and recognizing the influence of global capital flows (investment in technology startups), regulatory frameworks (such as data privacy laws), and technological advancements (like blockchain technology). These interactions are rooted in the broader historical context of financial globalization and digital transformation, which have enabled the rise of fintech as a distinct global field (Arner et al., 2016). The identification of this configuration is dynamic, evolving as new actors, regulations, and technologies emerge.

#### 4.2.2 Step 2: mapping field dynamics

The second step involves mapping the dynamics of the configurational field. This step focuses on analyzing the structural and relational features of the field, with particular attention to the distribution of what can be termed configurational capital. It is essential to consider both the objective structures—such as formal positions and hierarchies within the field—and the subjective perceptions of the actors involved. These perceptions influence how actors navigate and interpret the field, thereby shaping its dynamics. Once actors begin interacting within a configuration, they develop subjective perceptions of self-interest, shaped by their habitus. As they engage in these interactions, compromises and agreements are made, leading to the establishment of common cognitive structures among the actors. These cognitive structures define the types of capital that are valuable, their conversion rates, and the rules governing interactions within the field. This process results in the stabilization of positions, the formation of social closure, and the establishment of boundaries and hierarchies within the configurational field. In this process, habitus—characterized by field-specific traits and unique structuring—also contributes to the stability or instability of subjective and objective relations and aspects within a field, making their identification particularly significant.

For example, in the global renewable energy configuration, the field’s dynamics are shaped by interactions among governments, energy companies, environmental organizations, and consumers. Key forms of capital—economic capital (investment in renewable energy infrastructure), symbolic capital (global leadership in sustainability), and social capital (international partnerships)—define the interactions within this field. Mapping these dynamics requires analyzing the complex relationships among these actors, such as how the European Union’s renewable energy policies influence the strategies of global energy companies. Subjective perceptions, including public trust in renewable energy and corporate branding around sustainability, also impact how capital is valued and converted. This interplay stabilizes positions, such as the dominance of European and Chinese companies in renewable technologies, and establishes new hierarchies within the global energy sector (Sovacool and Geels, 2016).

#### 4.2.3 Step 3: assessing contextual influence

The third step in CFA involves assessing the influence of historical and cultural contexts on the dynamics of the configurational field. Fields are not isolated entities; they are embedded within broader socio-historical contexts that profoundly shape their formation, persistence, and transformation. This step requires examining both the internal relationships within the field and its external connections to other configurational fields.

For example, in the global fashion industry, the legacy of colonialism and the historical dominance of Western fashion houses continue to shape contemporary power dynamics. The rise of fast fashion, which relies on global supply chains and production in countries like Bangladesh and Vietnam, has altered traditional field dynamics. However, these shifts are embedded in a broader context of global labor inequalities and cultural hegemonies that continue to favor Western brands in terms of symbolic capital. Western fashion houses still dominate the global narrative of style and prestige, while the labor behind fast fashion remains undervalued in global supply chains, revealing the persistent influence of historical power imbalances (Crane, 2012). By incorporating these historical and cultural layers, CFA provides a nuanced understanding of how fields evolve in response to long-term societal changes and shifts in global power dynamics.

#### 4.2.4 Step 4: monitoring transformation

The final step in CFA is to monitor the transformation of the configurational field and its various components. This involves tracking changes in key parameters—field dynamics, capital distribution, and habitus—over time. Configurational fields are not static; they continuously evolve in response to external pressures, internal conflicts, and broader socio-historical transformations. Longitudinal data analysis is essential for capturing these changes and identifying the factors driving the field’s evolution.

For instance, in the global digital economy configuration, researchers might monitor the impact of emerging technologies such as artificial intelligence (AI) and big data analytics on traditional industries. AI is transforming fields ranging from healthcare to finance, challenging existing power structures and creating new opportunities for capital accumulation. Monitoring these transformations involves analyzing how major technology firms (e.g., Google, Amazon) leverage economic capital (investments in AI research) and symbolic capital (AI leadership) to reshape the global digital economy. Additionally, AI-driven changes in labor markets and the creation of new forms of expertise require careful observation to understand the broader societal impacts. By tracking these developments over time, researchers can gain insights into the long-term processes that reshape social fields and their configurations (Zuboff, 2019).

The three key analytical components in Configurational Fields Analysis (CFA) are the configurational field, capital, and habitus, which together form the foundational structure for analyzing various aspects of objects of inquiry in CFA. Analytically and methodologically, the identification and analysis of the different elements of these three concepts, as revisited in CFA, will illuminate various facets of configurations at different analytical stages. Particularly in the second stage of CFA, identifying these central concepts is essential for analytically determining the fundamental nature of configurations. Therefore, a precise understanding of their reconstruction in CFA is crucial. Moving forward, this analysis will employ Philip Gorski’s schematic model of Pierre Bourdieu’s field theory to constructively interrelate and analyze these dimensions.

### 4.3 Configurational field

The concept of the configurational field in CFA builds on Pierre Bourdieu’s theory of fields, with an added emphasis on the dynamic and contingent nature of social spaces (Bourdieu and Wacquant, 1992). Configurational fields are shaped by both objective structures—such as external rules, positions, and relationships that exist independently of individual actors—and subjective dynamics, which include the strategies, perceptions, and actions of those actors (Bourdieu, 1985). As discussed, these fields are not static entities but continually evolve as actors interact with one another, engage with surrounding structures, and respond to historical and cultural contexts (Emirbayer and Johnson, 2008). Drawing on Gorski’s schematic model, the following analysis will address these dual aspects by exploring how they shape the genesis, autonomy, heteronomy, size, shape, and boundaries of fields (Gorski, 2013). This examination will emphasize how historical and cultural factors influence the evolution and transformation of social fields, highlighting the complex interplay between structure and agency (Swartz, 1997; Calhoun, 2011).

#### 4.3.1 Objective structures: force field analogy

In Configurational Field Analysis (CFA), objective structures are conceptualized as configurational force fields—dynamic and relational networks of interdependent elements that shape and are shaped by actors within them. Diverging from Bourdieu’s original notion of fields as relatively stable structures with predefined rules and hierarchies, CFA redefines these structures as fluid, contingent spaces where boundaries, power relations, and capital flows are constantly reconstituted through actors’ interactions and historical contingencies (Bourdieu, 1985; Swartz, 1997). In this view, objective structures function not as fixed rules of competition but as adaptable configurations where forces such as capital distributions, regulatory frameworks, and socio-political shifts dynamically influence actors’ positions and opportunities (Jong, 2023b). The force field analogy within CFA emphasizes that these structures are neither stable nor universally applicable but vary across temporal, regional, and situational contexts, reflecting a field’s relational and indeterminate essence. Here, objective structures manifest as mutable conditions of possibility—complex arenas in which actors continually navigate and reshape the hierarchical and power-laden terrain through strategic actions, thereby reinforcing or transforming the field itself. This post-foundational reconceptualization aligns with CFA’s commitment to indeterminacy and contextual specificity, offering a flexible framework to capture the fluid nature of global and transnational fields, where the interplay of regulatory bodies, capital flows, and power dynamics is perpetually negotiated rather than statically imposed (Jong, 2024; Beck, 2016; Emirbayer and Johnson, 2008).

For example, the global finance sector illustrates how objective structures, such as regulatory frameworks, central banking policies, and international trade agreements, establish a force field within which financial institutions operate. Regulatory bodies like the Securities and Exchange Commission (SEC) in the U.S. or the Financial Conduct Authority (FCA) in the UK create fixed conditions that shape financial market dynamics, influencing the behavior of major banks, investment firms, and multinational corporations (Hall and Soskice, 2001). The force field established by these regulatory frameworks dictates the flow of capital, the allocation of risk, and the hierarchies of financial power, which institutions must strategically navigate to maintain competitiveness. These objective structures are not merely background constraints; they actively define the possibilities and limitations of actors’ strategies, much like physical forces determining the movement of objects within a field. This framework underscores how power dynamics and material conditions within fields are deeply embedded in structural contexts that actors must continually respond to and adapt to Fligstein (2001b).

#### 4.3.2 Subjective dynamics: playing field analogy

In CFA, subjective dynamics are understood as the process through which actors co-create and reshape the field itself, extending beyond strategies within a fixed structure to encompass the ways in which actors actively engage with, interpret, and transform the field’s evolving norms, hierarchies, and distributions of capital (Bourdieu, 1985; Emirbayer and Johnson, 2008). CFA views fields as “playing fields” where actors are not passive recipients of structural constraints but are reflexive agents who strategically navigate and redefine these structures and rules of games based on their specific relational understanding of the field (Swartz, 1997). Unlike Bourdieu’s notion of agency primarily influenced by habitus, CFA emphasizes that actors consciously assess and respond to the field’s fluid, relational nature, employing adaptive strategies that not only aim to maximize their positions but also to influence and negotiate the field’s structure itself. Configurational playing fields thus emerge as sites of continuous negotiation, where subjective dynamics transform fields into arenas of dynamic interaction; here, actors redefine their objectives, adapt to the actions of others, and either align with or disrupt existing power structures. This perspective moves beyond a static view of agency, positioning actors as active participants who engage with structural conditions, interpret relational opportunities, and seek to both exploit and reshape the existing configurations of power and influence within the field (Emirbayer and Johnson, 2008; Bourdieu, 1985; Swartz, 1997).

An example of this can be seen in the global technology sector, where companies like Apple, Google, and Amazon continuously develop strategies to navigate regulatory policies, market competition, and technological advancements. In the smartphone market, for instance, Apple’s strategy of creating a closed ecosystem—characterized by proprietary hardware and software—reflects its subjective response to both competitive pressures and regulatory challenges. This strategic choice not only allows the company to maintain control over its products but also maximizes its influence within the broader technology field, enabling it to shape consumer expectations and industry standards. Meanwhile, other tech companies may adopt different strategies, such as open-source platforms or aggressive pricing models, based on their perceptions of market opportunities and the actions of key competitors (Cusumano et al., 2015). These subjective dynamics underscore the continuous interaction between structure and agency, as actors oscillate between conforming to established rules and attempting to reshape the field to suit their strategic goals (Bourdieu and Wacquant, 1992).

#### 4.3.3 Field changes: genesis, autonomy, and heteronomy

##### 4.3.3.1 Genesis: emergence of new fields

The genesis of a field involves both objective genesis, which refers to the structural differentiation and autonomy of a new field (the condition of the possibility), and subjective genesis, which encompasses the strategic actions of actors that create new spaces and relations (desires and interests) (Gorski, 2013). In CFA, the genesis of a field is reconceptualized as a process of configurational coalescence, a dynamic and relational formation rather than merely a structural differentiation or a product of actor-driven construction. Moving beyond Bourdieu’s concept of field emergence, which emphasizes structural autonomy and actor participation as primary mechanisms, CFA situates genesis within a framework of fluid interdependencies where structural potentials and actor agency continually interact to shape new spaces of relations. This configurational approach to genesis recognizes the emergence of a field as contingent upon a specific convergence of factors—such as technological advancements, socio-economic transformations, and regulatory shifts—each of which interacts with the strategic actions of actors to form a complex, temporally bound, and context-sensitive relational space. This perspective emphasizes that fields do not emerge in isolation; rather, they are co-constituted through the entangled processes of structural opportunities and the actors’ desires, interests, and interventions. By framing field genesis as configurational coalescence, CFA highlights the provisional and evolving nature of fields, capturing the dynamic interplay that both enables and limits the formation of new, operative spaces within broader social configurations.

This reconceptualization acknowledges that fields are not pre-structured domains waiting for actors but are actively constituted through the alignment of structural and relational configurations. The case of cryptocurrency exemplifies this coalescent emergence. While blockchain technology provides the technical foundation (objective condition) that allows for a decentralized financial network, the cryptocurrency field did not materialize merely as a result of this innovation. Instead, it emerged through a deliberate and multifaceted configuration: developers, investors, and ideologically driven advocates collectively interpreted, promoted, and engaged with the potential of decentralized finance. Their actions established a unique space of economic and cultural relations within which new forms of capital (e.g., cryptocurrencies like Bitcoin and Ethereum) could circulate and redefine conventional notions of value, authority, and exchange. This process underscores that the genesis of configurational fields is marked by relational convergence, where structural opportunities and actor-driven strategies converge to produce a new, yet continually evolving, relational space (Swartz, 2020; Fligstein, 2001a, 2001b).

##### 4.3.3.2 Autonomy and heteronomy: internal logic vs. external influences

In CFA, autonomy and heteronomy are reframed as relationally dynamic states rather than fixed attributes of a field. Traditional field theory defines autonomy as a field’s capacity to sustain its internal logic and priorities independently, while heteronomy refers to the degree of influence exerted by external forces that shape or distort the field’s internal dynamics (Bourdieu and Wacquant, 1992). In CFA, however, autonomy is seen not as an absolute independence from external pressures but as a field’s capacity to assert and adapt its unique logics amidst external influences. Heteronomy, in turn, reflects the extent to which a field’s norms, practices, and structures are permeated and reshaped by external forces. This reconceptualization treats autonomy and heteronomy as fluid, interdependent conditions that are continually renegotiated within a field’s interactions with its surrounding configurations. Fields do not operate in isolation but exist within an interconnected social landscape, where they must actively reassert or transform their internal structures to accommodate, resist, or integrate pressures from adjacent fields and broader socio-political forces. In this way, autonomy and heteronomy are not binary states but dynamic equilibria shaped by the ongoing relational processes through which fields navigate, absorb, and sometimes redefine external influences within their evolving configurations (Swartz, 1997; Bourdieu and Wacquant, 1992).

For instance, the higher education field exemplifies this configurational balancing act. Traditionally, universities held substantial autonomy, governed by principles such as academic freedom, peer review, and scholarly integrity. However, neoliberal shifts and global competition have intensified external pressures, leading to a heightened state of heteronomy. Policies that prioritize measurable outputs, market competitiveness, and rankings demand that universities reshape their practices, often reorienting institutional goals toward economic viability and quantifiable success metrics rather than intellectual exploration. In response, universities engage in a strategic reconfiguration, balancing their commitment to academic values with the need to integrate economic imperatives. This process reflects CFA’s emphasis on autonomy and heteronomy as not fixed qualities but evolving relational states that configure how fields maintain integrity and coherence while adapting to external demands. Here, autonomy is not a resistance to heteronomy but a recalibration of field-specific priorities to accommodate, negotiate, and reinterpret external influences (Marginson, 2016; Gorski, 2013).

#### 4.3.4 Size, shape, and boundaries: defining the field’s dimensions

##### 4.3.4.1 Size: expansion and contraction of fields

The size of a field refers to the total population of positions within it, with changes in size indicating the expansion or contraction of influence and resources (Bourdieu and Wacquant, 1992). This dynamic is shaped by both objective conditions, such as resource availability and external opportunities, and subjective factors, such as the strategic actions of actors seeking to expand or protect their positions (Swartz, 1997). In CFA, the “size” of a field reflects more than just the number of actors or positions within it; it denotes the field’s dynamic capacity to expand or contract based on shifts in relational influence, resources, and external opportunities. Unlike in Bourdieu’s framework, where size might imply a relatively static metric of inclusion, the configurational approach emphasizes size as a fluid outcome of strategic actions and structural opportunities. Changes in field size are both contingent upon material conditions—such as technological resources, regulatory shifts, or economic incentives—and the active strategies of actors seeking to either expand their reach or consolidate power within an evolving network of relations (Bourdieu and Wacquant, 1992; Swartz, 1997).

The renewable energy sector exemplifies this configurational perspective on field size. Structural conditions, such as governmental subsidies, global environmental policies, and technological advancements in renewable technologies, create a fertile ground for expansion. Yet, this growth is equally driven by strategic actions from key actors—such as companies investing in solar and wind technologies, countries committing to renewable energy targets, and NGOs advocating for sustainability. Together, these factors do not merely increase the number of positions but reshape the network of relational influence within the energy sector, altering the field’s overall configuration. This continuous redefinition of field size underscores how expansion and contraction reflect a recalibration of resources, power dynamics, and strategic investments in response to changing environmental, economic, and political landscapes (Sovacool, 2016).

##### 4.3.4.2 Shape: hierarchies and orthodoxy

The shape of a field reflects its hierarchy and orthodoxy, with more hierarchical fields exhibiting significant power differences and orthodox fields adhering to a central set of beliefs or practices. The shape of a field is influenced by both objective structures (e.g., institutional frameworks) and subjective dynamics (e.g., actors reinforcing or challenging the hierarchy) (Gorski, 2013). In CFA, the shape of a field represents a dynamic relational structure defined by its hierarchies and orthodoxy, which together delineate dominant positions and shared practices. Unlike Bourdieu’s perspective, which treats hierarchy and orthodoxy as stable elements preserving a field’s internal order, CFA views the shape of a field as a mutable configuration where power structures and norms are continuously reinforced, contested, and redefined. Hierarchies within a configurational field are not merely rigid, top-down systems; they are relational outcomes of actors’ strategic interactions as they seek to assert dominance, uphold or challenge prevailing norms, or introduce new practices. This configurational approach captures the fluidity of a field’s shape, acknowledging that its hierarchy and orthodoxy emerge through ongoing negotiations between objective structures—such as institutional rules—and subjective dynamics, including actors’ efforts to align with or disrupt established power relations.

For example, in the global luxury goods market, the shape of the field is sharply hierarchical, with a few dominant brands, like Louis Vuitton and Chanel, occupying positions of concentrated economic and symbolic capital. These brands embody the field’s orthodoxy, characterized by exclusivity, heritage, and craftsmanship, which sets the standards for what is considered “luxury.” Smaller brands or new entrants in the field must navigate this hierarchy, often by either adhering to this orthodoxy to gain legitimacy or by strategically innovating to carve out alternative niches. Thus, the field’s shape is actively shaped by the tension between conformity to and divergence from established norms, revealing a configurational structure where power relations and field norms are constantly reinterpreted and renegotiated by actors at various positions within the hierarchy (Hoffmann and Coste-Manière, 2012).

##### 4.3.4.3 Boundaries: defining inclusion and exclusion

In CFA, field boundaries are conceptualized as dynamic zones of negotiation rather than fixed limits, continually redefined by both internal dynamics and external pressures. Diverging from Bourdieu’s view of boundaries as stable demarcations that preserve a field’s autonomy, CFA treats boundaries as permeable and contestable relational spaces where inclusion and exclusion are fluid outcomes shaped by strategic maneuvers, alliances, and external interventions. Boundary shifts are configurational outcomes, reflecting the evolving access to resources, influence, and opportunities within the field. These changes may occur as zero-sum adjustments—where one field’s expansion limits another’s—or as non-zero-sum transformations, in which new relational spaces form without displacing existing structures. This understanding of boundaries highlights their mutable nature, emphasizing that a field’s inclusivity or exclusivity is continuously negotiated and contingent upon the broader configurations and power dynamics that permeate and reshape it (Bourdieu and Wacquant, 1992; Gorski, 2013).

An example of zero-sum boundary changes can be observed in the tech industry, where the rise of digital streaming services like Netflix and Amazon Prime has expanded the boundaries of the entertainment field while simultaneously shrinking the space occupied by traditional television networks. Conversely, non-zero-sum boundary changes can be seen in the biotechnology field, where the inclusion of new actors such as genetic research startups and synthetic biology firms has expanded the field without necessarily displacing established pharmaceutical companies (Thacker, 2005).

The permeability of these boundaries is shaped by objective conditions, such as regulatory frameworks and resource availability, while subjective strategies involve actors either fortifying boundaries to protect their positions or relaxing them to allow new entrants, depending on their goals within the field (Emirbayer and Johnson, 2008). For example, established pharmaceutical companies may fortify boundaries through patent protections, while startups might advocate for regulatory flexibility to increase inclusion within the field. These dynamic processes reveal how field boundaries are continually contested and renegotiated, reflecting the ongoing struggle between inclusion and exclusion.

### 4.4 Configurational capital

The concept of configurational capital in Configurational Field Analysis (CFA) is based on Bourdieu’s notion of capital but emphasizes the dynamic, contingent, and relational aspects of how resources and assets are utilized within fields (Bourdieu, 1986). Configurational capital encompasses various forms of resources—economic, cultural, social, symbolic, religious, and more—that actors use to gain power and influence within dynamic fields (Swartz, 1997; Calhoun, 2011). This reconfiguration is contingent on the interplay of objective structures, historical contexts, and subjective perceptions, making capital an adaptive and strategic force within the relational and contingent logic of CFA (Emirbayer and Johnson, 2008; Gorski, 2013). Configurational capital thus transcends the static accumulation of assets, focusing instead on their contextual utility and transformation. It highlights how power is negotiated through the strategic valuation, conversion, and circulation of resources, reflecting the dynamic interplay between field structures and individual agency. This section will explore these dimensions, integrating both the objective and subjective aspects of configurational capital, with a focus on the dynamics of capital exchange, autonomy, heteronomy, size, circulation, and hierarchy (Gorski, 2013).

#### 4.4.1 Objective structures: configurational capital and power

Configurational capital represents a fluid and relationally determined array of resources that actors accumulate, convert, and deploy to influence and shape social fields. Departing from Bourdieu’s relatively fixed categories of capital—economic, cultural, social, and symbolic—CFA reinterprets these forms of capital as adaptable entities whose value and utility are contingent on the field’s evolving configurations (Bourdieu, 1986; Swartz, 1997). In fields such as business and politics, economic capital remains pivotal, with financial resources functioning as a direct source of decision-making power. Corporate campaign donations and lobbying in the political field illustrate how economic capital shapes policy and electoral outcomes (Gorski, 2013). However, in fields such as academia, art, or media, cultural and social capital take on heightened significance. For instance, in academia, cultural capital—embodied in credentials, publications, and institutional affiliations—determines recognition and authority, while social capital, reflected in professional networks, amplifies influence within the scholarly community (Maton, 2008; Grenfell, 2012).

A distinctive feature of configurational capital in CFA is its adaptability; its value is relationally and contextually determined. This adaptability is evident in the conversion of one form of capital into another, such as the use of cultural capital (e.g., academic achievements) to build social capital through strategic collaborations, which may ultimately yield economic rewards, such as research grants or promotions. The ability to convert and recalibrate capital forms is a key driver of power and influence within fields, as actors navigate shifting relational dynamics and capitalize on emergent opportunities (Crossley, 2001). By emphasizing capital as a relational and dynamic resource, CFA underscores its role not merely as an asset but as a force that reflects and reproduces the relational hierarchies of fields. This perspective reveals that power in CFA is not rooted in the static possession of resources but in the continuous and strategic reconfiguration of their value and utility within an evolving structural landscape (Emirbayer and Johnson, 2008).

#### 4.4.2 Subjective dynamics: capital conversion and perception

Configurational capital is also shaped by subjective dynamics, emphasizing how actors interpret, assess, and convert capital based on their perceptions of its value within specific relational contexts. Unlike static conceptualizations, CFA highlights the fluidity of capital conversion, viewing it as a contextual recalibration that depends on strategic agency and field-specific opportunities (Bourdieu, 1986; Swartz, 1997). For example, in the art world, cultural capital, such as artistic acclaim or critical recognition, can be strategically transformed into economic capital. When an artist’s work is featured in prestigious exhibitions or receives awards, its market value increases, illustrating the conversion of symbolic recognition into financial gain (Bourdieu, 1993). Similarly, in academia, scholars may leverage cultural capital (e.g., high-impact publications) to build social capital through collaborations, which can lead to access to funding or professional advancement (Reay, 2004).

Through the lens of CFA, capital conversion is not merely transactional but deeply contextual and relational. Actors engage in strategic calculations, interpreting the evolving relational landscape of their field to maximize the value of their resources. For instance, a corporate executive might use social capital (professional networks) to navigate a merger by aligning with influential stakeholders, thereby converting relational influence into economic or symbolic gains. This strategic adaptability highlights how subjective perceptions and field-specific dynamics shape the interplay of capital forms, underscoring the relational logic of CFA (Grenfell, 2012). Configurational capital’s subjective dimension reveals the active role of agency in capital dynamics. Actors do not passively accumulate resources; they continuously reinterpret and recalibrate their capital based on emergent opportunities and constraints within their fields. This process underscores the adaptability and resilience of configurational capital, positioning it as a cornerstone of strategic action and relational power in CFA (Emirbayer and Johnson, 2008).

#### 4.4.3 Field-specific dynamics: capital exchange rates and autonomy

##### 4.4.3.1 Exchange rates: value fluctuations and power shifts

In CFA, exchange rates between various forms of capital are dynamic, reflecting the shifting power relations and competitive landscapes within and across fields. Unlike static valuations, CFA treats these exchange rates as contingent and relational, adjusting in response to both objective factors—such as market demands, regulatory changes, and scarcity of resources—and subjective factors, including actors’ interpretations of legitimacy, prestige, and symbolic value (Bourdieu, 1986; Swartz, 1997). These fluctuating exchange rates underscore that capital’s worth and convertibility are continuously redefined by the interplay between actors’ strategic positioning and broader structural conditions, emphasizing that capital’s value is not intrinsic but contextually negotiated. For example, in academia, cultural capital—embodied in prestigious positions or high-impact publications—fluctuates in value based on shifts within the field. When policy and funding priorities shift toward areas like artificial intelligence, scholars with expertise in AI find their cultural capital newly convertible into economic capital, accessing greater grant opportunities and higher salaries. Such fluctuations reflect a recalibration of capital value within the field, enabling actors with newly prioritized forms of capital to enhance their influence and alter the field’s internal hierarchy (Emirbayer and Johnson, 2008).

Similarly, in the global tech industry, the ascent of intellectual property (IP) and data as valuable forms of economic capital has dramatically reshaped power dynamics. Companies like Google, Amazon, and Facebook accumulate vast data resources, converting this data into economic capital through targeted advertising and market control, and into symbolic capital by positioning themselves as technological leaders. This shift in capital exchange rates exemplifies how technological advancements can redefine power structures within a field, elevating certain actors while diminishing others’ influence based on their capital adaptability and alignment with emerging market dynamics (Zuboff, 2019). Through CFA’s lens, these shifting exchange rates capture the fluid reconfiguration of capital, as actors leverage evolving relational dependencies and strategic maneuvers to gain or maintain power within complex, interconnected fields.

##### 4.4.3.2 Autonomy: stability of capital within fields

In CFA, autonomy is defined by the stability and resilience of a field’s specific forms of capital, which retain their value and internal logic despite external pressures and interference. Unlike fields more susceptible to external influences, highly autonomous fields possess a well-defined hierarchy of capital that is insulated from external forces, allowing them to uphold distinct norms, standards, and internal structures (Bourdieu and Wacquant, 1992; Swartz, 1997). This stability reinforces a field’s unique identity, ensuring that its forms of capital remain valuable and respected even amid shifting economic, political, or cultural environments. The legal field in many Western contexts exemplifies this high degree of autonomy. Its capital—such as legal expertise, credentials, and professional reputation—retains value and legitimacy independently of external political or corporate pressures. For instance, the judiciary, a cornerstone of the legal field, often resists political influence to safeguard legal capital, maintaining ethical standards, jurisprudential principles, and judicial independence. This autonomy enables the legal field to preserve the integrity of its capital, such as the rule of law, even when confronted by external interests, thus ensuring that its internal hierarchy and standards remain intact (Gorski, 2013).

Similarly, the academic field demonstrates autonomy through the stable valuation of peer-reviewed publications and scholarly credentials as core forms of cultural capital. Despite external pressures, such as shifting government funding priorities or increasing corporate involvement, the academic field defends its standards for knowledge production and dissemination. Peer-reviewed research, as a capital form, underscores the field’s resistance to external pressures, allowing it to preserve a rigorous internal logic that prioritizes scholarly credibility and intellectual independence (Berman, 2012). By insulating its internal dynamics, an autonomous field maintains a stable and coherent hierarchy of capital, enabling it to resist external distortions and sustain its distinctive characteristics and standards.

#### 4.4.4 Heteronomy and foreign capital: external dependencies and symbolic subordination

##### 4.4.4.1 Heteronomy: external dependencies and distortions

In CFA, heteronomy represents the field’s vulnerability to external forces that reshape its internal priorities, hierarchies, and operational logic by imposing dependencies on resources or forms of capital originating from other fields. Unlike a traditional view where fields are assumed to possess distinct boundaries that guard their autonomy, CFA posits that fields are inherently interconnected and susceptible to configurational imbalances when external capital exerts undue influence. Heteronomy, therefore, signifies the loss of a field’s configurational integrity as it becomes increasingly subject to external pressures, leading to a reorientation of its core values, practices, and relational dynamics (Bourdieu and Wacquant, 1992; Swartz, 1997). For instance, the media industry illustrates how heteronomy materializes through its growing dependency on advertising revenue and political patronage. Traditionally, journalistic capital, grounded in public trust, investigative rigor, and editorial independence, defined the media field’s unique logic and sustained its autonomy. However, as media outlets become increasingly reliant on advertising dollars and political alliances, the internal criteria of value within the field shift, distorting the journalistic function. Sensationalism, content geared toward advertiser preferences, and politically aligned reporting begin to supplant unbiased, in-depth analysis. This reconfiguration of values and priorities does not merely alter the media’s practices but also reorganizes its relational hierarchies—elevating actors who secure external capital and diminishing those dedicated solely to journalistic integrity (Gorski, 2013).

Through CFA’s lens, heteronomy is not a mere encroachment of external influences but a configurational shift where the influx of external capital recasts the field’s power structures, relational dependencies, and definitional boundaries. This process destabilizes the field’s autonomy by embedding external agendas within its internal operations, thereby aligning the field’s activities with external priorities at the expense of its original mandate. Consequently, CFA reveals that heteronomy induces a dynamic and potentially irreversible reconfiguration of the field’s structure, altering the value, utility, and impact of capital within it and reinforcing dependencies that further entrench external distortions in the field’s functioning. In the political field, for instance, the reliance on economic capital for campaign financing has introduced significant heteronomy. Political actors who depend on corporate donations or wealthy individuals to fund their campaigns may find their policies influenced by these external sources of capital. This reliance distorts the internal logic of the political field, where democratic representation and public service are supposed to be the primary drivers, by subordinating these principles to the demands of external economic interests (Crouch, 2011). These examples highlight how external dependencies can erode the autonomy of a field, distorting its internal structure and diminishing the value of its traditional forms of capital.

##### 4.4.4.2 Subjective heteronomy: symbolic subordination

In CFA, subjective heteronomy reflects a field’s symbolic vulnerability when external symbolic logics permeate its internal structures, influencing how legitimacy, authority, and value are perceived within the field. Unlike overt heteronomy, which is driven by material dependencies, subjective heteronomy involves a subtler form of symbolic subordination, where the field’s own symbolic capital—its values, norms, and standards—becomes subordinated to external symbolic values, reorienting perceptions of legitimacy and altering the internal dynamics and hierarchies of the field (Bourdieu, 1993). This infiltration of external symbolic logics subtly shifts the field’s internal valuation processes, as actors increasingly assess their capital and status against externally imposed norms rather than the field’s original criteria. For example, in academia, subjective heteronomy may occur when commercial or political metrics—such as market-driven measures of research impact or politically motivated funding priorities—begin to influence what is considered legitimate scholarly capital. Over time, this reorients academic hierarchies, favoring those who align with external symbols of value, such as citation counts or fundability, over traditionally autonomous markers of academic rigor or intellectual independence.

Through the lens of CFA, subjective heteronomy represents a configurational reorientation where external symbolic values infiltrate and reshape the field’s internal hierarchies, gradually recalibrating its standards of legitimacy. This process not only affects how capital is perceived but also how actors within the field strategize, increasingly aligning their practices and objectives with these external symbolic logics to maintain or enhance their status. Ultimately, subjective heteronomy destabilizes the field’s symbolic autonomy, as its internal logic is redefined in alignment with external expectations, creating a dependency on external validation that reconfigures both the field’s relational dynamics and its core identity. For example, in the field of higher education, universities have traditionally valued academic capital, such as scholarly research and teaching excellence. However, as external symbolic logics—such as global university rankings and market-driven metrics—gain prominence, they begin to dictate what is deemed legitimate and valuable within the field. Universities increasingly prioritize rankings, employability scores, and revenue from international students, which can devalue traditional academic capital in favor of external validation. This shift alters the internal logic of the field, leading to a reconfiguration of its hierarchy, where those who excel in metrics aligned with external symbolic logics gain prominence, potentially at the expense of scholarly rigor and educational integrity (Gorski, 2013). The infiltration of these external values into higher education exemplifies how subjective heteronomy can destabilize a field’s autonomy and disrupt its internal structures by devaluing its traditional forms of capital.

#### 4.4.5 Size, circulation, and boundary changes: capital flow and negotiability

##### 4.4.5.1 Size and circulation: measuring field influence

In CFA, size and circulation reflect a field’s influence by examining the extent to which its specific forms of capital permeate and reshape adjacent fields and social configurations. Unlike a static measure of size, CFA treats the circulation and reach of capital—such as technological expertise, data resources, or cultural prestige—as dynamic indicators of a field’s expansion and societal impact (Bourdieu and Wacquant, 1992). As capital flows outward, it magnifies the field’s influence, integrating its logic and resources into other fields and affecting broader social norms and practices (Swartz, 1997). The global tech industry, especially through innovations in big data and AI, exemplifies this expansive reach. Here, capital such as technological knowledge, data ownership, and AI capabilities circulates widely, embedding tech-driven values and frameworks within fields like healthcare, finance, and governance. This expansive circulation underscores the field’s considerable influence, as actors like Google, Amazon, and Microsoft leverage their control over critical resources to shape practices and power structures beyond the tech industry itself. Such circulation not only reinforces the dominance of these leading actors within the tech field but also reconfigures hierarchies and operational standards across interconnected fields. In CFA terms, this inter-field circulation of capital highlights the link between a field’s size—defined by its reach and resource control—and its capacity to shape larger societal configurations, underscoring how the influence of dominant actors extends far beyond their immediate domain (Emirbayer and Johnson, 2008).

##### 4.4.5.2 Boundary changes and negotiability: capital exchange and symbolic conflict

In CFA, boundary changes and negotiability within and between fields occur when shifts in the permeability and directionality of capital exchange redefine how fields interact, restructure hierarchies, and influence each other’s internal dynamics. Unlike static boundaries, CFA views these borders as flexible zones of interaction shaped by both objective changes—such as regulatory adjustments that facilitate capital flow—and subjective conflicts over the symbolic or practical value of diverse forms of capital (Bourdieu and Wacquant, 1992; Swartz, 1997). A prominent example is seen in the convergence of the tech and financial fields with the rise of fintech, where regulatory reforms have made field boundaries more permeable, enabling the flow of technological and economic capital across previously distinct domains. This permeability allows tech companies like PayPal and Square to leverage their technological capital, challenging traditional banks and reshaping financial services through digital innovation. In CFA terms, these boundary shifts are not merely about capital flow but signify symbolic conflicts as new actors redefine value, influence, and authority within and across fields. Such transformations underscore that boundary changes do not only alter the power dynamics within individual fields but also have wider social implications, creating new structures and hierarchies that ripple across interconnected fields. This reconfigurational process reveals that boundary shifts are a potent force in reshaping fields and the broader social landscape, reflecting how capital exchange and symbolic competition drive structural and relational evolution in today’s complex, interconnected social configurations (Gorski, 2013).

### 4.5 Configurational habitus

The concept of configurational habitus within Configurational Field Analysis (CFA) reimagines Bourdieu’s original idea of habitus as a dynamic and relational construct. Traditional interpretations of habitus emphasize its role as a set of enduring dispositions that align individuals with the structures and logics of specific fields (Bourdieu, 1990). However, this perspective often underestimates the complexity, fluidity, and relationality inherent in modern globalized social configurations. Configurational habitus addresses these limitations by embedding habitus within a relational framework that recognizes the co-constitution of stability and adaptability, structure and agency, and local and transnational dynamics (Wacquant, 2016; Decoteau, 2016). It is not merely a bridge between individual dispositions and structural fields; it is an active mechanism that reflects and reshapes the evolving relational dynamics of social configurations (Hayes, 2020). This section advances the concept of habitus by integrating insights into reflexivity, pluralism, and the interplay of objective and subjective forces, positioning it as a key element of CFA’s analytical framework (Mouzelis, 2008). Configurational habitus provides a lens for understanding how individuals navigate, sustain, and transform the fluid and interconnected landscapes of modern social life.

#### 4.5.1 Objective structures: durable and transposable dispositions

At its core, configurational habitus is deeply rooted in objective structures that provide continuity and coherence within social configurations. However, unlike the rigid, bounded structures of traditional field theory, these objective elements are reinterpreted as relational and processual, evolving through interactions across overlapping configurations (Burnett and Veenstra, 2017; Peters, 2013). Objective structures—such as institutional norms, cultural hierarchies, and material distributions of power—serve as anchors for habitus. Yet, within a configurational framework, these structures are understood as contingent and historically situated. For instance, the academic field’s emphasis on peer-reviewed publications and institutional prestige operates as a durable structure. However, these norms are continuously renegotiated through global pressures, such as the rise of interdisciplinary research and the increasing influence of non-academic stakeholders (Grenfell, 2012; Wacquant, 2016). Configurational habitus reflects the interplay between these stable but relational structures and individual dispositions. It emphasizes the relational logic of configurations, where structures are co-constituted by their interactions within broader social and temporal contexts. By aligning individual practices with these shifting relational dynamics, configurational habitus ensures coherence and adaptability within social fields, challenging the static assumptions of traditional field theory.

#### 4.5.2 Subjective dynamics: contextual adaptability and reflexive action

Configurational habitus not only reflects the influence of objective structures but also incorporates subjective dynamics that empower individuals to engage reflexively with their social environments. This dual emphasis on structure and agency aligns with the broader relational framework of CFA, positioning configurational habitus as a mechanism for both continuity and transformation in social configurations (Decoteau, 2016; Mouzelis, 2008). Unlike deterministic interpretations that portray individuals as passive products of structural forces, configurational habitus highlights the active role of reflexivity. Reflexivity enables individuals to assess, recalibrate, and adapt their dispositions in response to shifting field dynamics. This reflexive adaptability becomes particularly significant in rapidly changing or contested fields, such as digital media or global finance, where the pace of transformation often outstrips the stability of traditional structures (Adams, 2006; Schirato and Webb, 2002).

For example, professionals in technology-driven fields must constantly update their skills and strategies to remain competitive. Configurational habitus equips these individuals with the capacity to interpret and navigate the relational shifts within their configurations. Through reflexive engagement, they integrate new competencies and reposition themselves within evolving hierarchies of power and influence. This dynamic adaptability illustrates how configurational habitus operates as a responsive mechanism, facilitating alignment with emergent field logics while preserving coherence with broader configurations. Configurational habitus also reflects the pluralistic nature of modern social life, where individuals often navigate multiple overlapping configurations. Reflexivity enables them to reconcile competing logics—such as those of professional, cultural, or transnational fields—by recalibrating their practices and dispositions to align with the relational dynamics of these intersecting contexts (Stone et al., 2012; Rafieian and Davis, 2016). This capacity for adaptive agency underscores the transformative potential of configurational habitus, allowing individuals to both sustain and reshape the relational structures that define their fields.

#### 4.5.3 Formation of configurational habitus: socialization and embodiment

The formation of configurational habitus emerges through intertwined processes of socialization and embodiment. This dual approach highlights how individuals internalize relational structures and develop dispositions that align with the dynamics of their configurations. Drawing on contemporary scholarship, this section reframes habitus formation as a dynamic and context-sensitive process that integrates cultural, material, and symbolic elements (Reay, 2004; Skeggs, 2004; Stone et al., 2012).

##### 4.5.3.1 Socialization across relational contexts

Socialization is central to the development of configurational habitus, embedding individuals within the power dynamics and relational logics of their social environments. Early socialization—shaped by family, education, and community—instills foundational dispositions that guide individuals’ interactions with various fields throughout their lives. However, within a configurational framework, these dispositions are not fixed; they evolve through continual engagement with shifting relational configurations (Burnett and Veenstra, 2017; Singh, 2022). For instance, children from affluent backgrounds may internalize dispositions aligned with the accumulation of cultural and economic capital, enabling them to navigate elite institutions and globalized professional fields with ease. Conversely, individuals from marginalized contexts develop dispositions that reflect the constraints and challenges of their environments, often cultivating adaptive strategies for navigating fields with limited resources (Reay, 2004; Moi, 1991; Setton, 2011). These processes reveal the relational and contingent nature of socialization, where configurational habitus is shaped by the interplay of historical, cultural, and structural forces.

##### 4.5.3.2 Embodiment of social distinctions

Beyond cognitive internalization, configurational habitus is profoundly embodied. It manifests in physical demeanor, aesthetic preferences, and social practices that signal an individual’s alignment with specific relational configurations (Bourdieu, 1984; Wacquant, 2016; Stewart and Fielding, 2022). Embodied dispositions serve as visible markers of social positioning, reinforcing distinctions within and across fields. For example, individuals navigating high-status professional fields often exhibit refined tastes and behaviors that align with dominant cultural norms, such as preferences for particular forms of art, cuisine, or attire (Skeggs, 2004; Crossley, 2001). These embodied practices not only signal alignment with specific configurations but also contribute to the reproduction of power dynamics by reinforcing field-specific hierarchies (Noble and Watkins, 2003). The embodiment of configurational habitus underscores its role in naturalizing social distinctions, making relational hierarchies appear intuitive and self-evident. Yet, these embodied practices are also sites of resistance and transformation. As individuals engage reflexively with their configurations, they may adopt, reject, or subvert embodied norms, reshaping the relational logics of their fields. This dual capacity for reproduction and innovation highlights the adaptive and relational nature of configurational habitus (Singh, 2022; Farnell, 2003).

#### 4.5.4 Role of configurational habitus in social fields: practical sense and social reproduction

Configurational habitus plays a dual role in social fields: it serves as both a mechanism for intuitive navigation and a driver of structural reproduction. This dual function reflects its capacity to mediate the relational dynamics of configurations, aligning individual practices with the implicit logics of fields while simultaneously reinforcing and reshaping their structures (Bourdieu, 1990; Maton, 2008).

##### 4.5.4.1 Practical sense: navigational reflexes in relational configurations

One of the defining features of configurational habitus is its practical sense—a tacit understanding that enables individuals to navigate complex and evolving social configurations. This intuitive “feel for the game” emerges through prolonged engagement with relational fields, allowing individuals to internalize the unwritten rules and power dynamics that shape their environments (Theiner and Fogle, 2018; Fligstein, 2001a). For example, professionals in high-pressure fields like finance or law often demonstrate an acute sensitivity to relational cues, such as shifts in authority or changes in market dynamics. These navigational reflexes enable them to act strategically within their configurations, seizing opportunities, mitigating risks, and maintaining their positions within competitive hierarchies (Crossley, 2001; Decoteau, 2016). Configurational habitus thus functions as a relational compass, guiding individuals through the fluid and interconnected landscapes of modern social life. However, this practical sense is not merely an individual skill; it reflects the broader relational dynamics of configurations. By aligning individual actions with the evolving logics of fields, configurational habitus contributes to the coherence and stability of social configurations. This dynamic underscores its relational and processual character, distinguishing it from static or deterministic interpretations of habitus (Lau, 2004).

##### 4.5.4.2 Social reproduction: configurational continuity and power dynamics

In addition to facilitating navigation, configurational habitus plays a crucial role in the reproduction of social structures. By aligning individual dispositions with the norms, values, and hierarchies of their fields, configurational habitus ensures the continuity of relational configurations over time (Bourdieu, 1990; Hunter, 2004). For instance, within the education system, students internalize field-specific dispositions, such as deference to authority and the pursuit of credentials. These dispositions not only shape their practices within the field but also perpetuate broader societal hierarchies, as individuals carry these ingrained behaviors into professional contexts (Reay, 2015; Edgerton and Roberts, 2014). Through this process, configurational habitus embeds the relational logics of fields into the actions and strategies of their participants, reinforcing existing power dynamics and sustaining the broader social order. Yet, this reproductive function is not absolute. Configurational habitus also incorporates the potential for resistance and transformation. As individuals engage reflexively with their configurations, they may challenge or subvert dominant norms, creating spaces for alternative practices and relational dynamics (Miller, 2016). This tension between continuity and change highlights the adaptive and relational nature of configurational habitus, positioning it as a key mechanism for both stability and transformation within social fields (Haluza-Delay, 2008).

#### 4.5.5 Dynamics of configurational habitus: reproduction, change, and crisis

Configurational habitus operates within a tension between stability and transformation, mediating the reproduction of social structures while enabling adaptive responses to evolving relational dynamics. This dual capacity reflects the dynamic and contingent nature of habitus as conceptualized within CFA, where continuity and change coexist in a constant interplay (Bourdieu, 1990; Gorski, 2013).

##### 4.5.5.1 Reproduction: configurational stability through durable dispositions

Configurational habitus inherently tends toward the reproduction of social structures by embedding durable dispositions that align individuals with the relational logics of their fields. This reproductive quality maintains stability within configurations, as individuals act in ways that reinforce established norms, hierarchies, and power dynamics. For example, professionals in elite institutions often internalize practices and values—such as exclusivity, competition, and deference to authority—that sustain the institutional and societal hierarchies they inhabit (Grenfell, 2012; Reay, 2004). These durable dispositions ensure that individuals not only navigate but also perpetuate the relational structures of their configurations, embedding their logics into daily practices and long-term strategies. However, the stability afforded by configurational habitus is not static; it evolves through incremental adjustments. These micro-adaptations reflect the cumulative effects of relational shifts, where subtle changes in field dynamics lead to gradual transformations in individual and collective practices (Gorski, 2013). This slow, layered process illustrates how configurational habitus sustains coherence within dynamic configurations while accommodating relational flux.

##### 4.5.5.2 Adaptive transformation: reflexive adjustment and relational realignment

Despite its reproductive tendencies, configurational habitus possesses an inherent capacity for adaptive transformation. This adaptability arises from its relational foundation, which enables individuals to recalibrate their dispositions in response to new configurations or shifts in field dynamics. For instance, migrants transitioning to different cultural or professional environments often adjust their habitus to align with the relational logics of their new contexts. These adjustments may involve adopting new linguistic, aesthetic, or strategic practices while retaining traces of their original dispositions (Skeggs, 2004; Wong and Liao, 2022). This dual process of assimilation and retention highlights the configurational nature of habitus, where change occurs within the continuity of relational structures. Moreover, configurational habitus fosters agency by enabling individuals to engage reflexively with their configurations. Reflexivity allows individuals to evaluate and adapt their practices strategically, negotiating power dynamics and relational constraints to achieve their objectives (Decoteau, 2016). This reflexive adaptability underscores the transformative potential of configurational habitus, positioning it as a mechanism for both personal and social innovation within dynamic fields.

##### 4.5.5.3 Crisis: dispositional disruption and reflexive reconfiguration

Crisis represents a critical juncture in the dynamics of configurational habitus, where profound shifts in field configurations disrupt the alignment between dispositions and relational structures. These disruptions reveal the contingent nature of habitus, prompting reflexive reconfiguration as individuals adapt to altered social realities. For example, economic upheavals or technological disruptions—such as the rise of automation—may render previously stable dispositions obsolete. Workers in traditional industries may find that their skill sets and strategies no longer align with the demands of reconfigured fields. In response, individuals must reassess and recalibrate their practices, acquiring new competencies or transitioning into different configurations entirely (Beck, 2000; Wacquant, 2016). Crisis highlights the dual role of configurational habitus as both a stabilizing and transformative force. While it aligns individuals with the relational logics of their configurations, it also possesses the flexibility to respond to external shocks, enabling individuals to navigate and shape emerging dynamics (Pop, 2007). This reflexive reconfiguration underscores the adaptability and resilience of configurational habitus, demonstrating its capacity to sustain coherence while facilitating transformation within relational fields.

#### 4.5.6 Homology, doxa, and crisis: structural alignment and transformation

In CFA, homology, doxa, and crisis are reimagined to reflect the relational and dynamic dimensions of configurational habitus. These concepts reveal how habitus aligns with, reinforces, or challenges relational structures within evolving configurations, emphasizing the adaptability and transformative potential of social practices.

##### 4.5.6.1 Homology: dynamic alignment with configurational structures

Homology in the context of configurational habitus refers to the alignment of dispositions with the relational structures and logics of a specific configuration. However, unlike static interpretations that treat homology as a fixed fit between habitus and field, CFA conceptualizes it as a dynamic alignment that evolves with shifts in relational dynamics (Maton, 2008; Grenfell, 2012). For example, professionals in the corporate sector may internalize competitive instincts, hierarchical respect, and efficiency-driven practices, aligning their habitus with the dominant values of the corporate field. This alignment allows them to navigate the field effectively, adapting to subtle changes in organizational structures and market demands. Configurational alignment thus serves as a stabilizing force, ensuring coherence within fields while accommodating relational fluidity (Swartz, 1997; Burnett and Veenstra, 2017). Moreover, homology is not merely a mechanism for continuity; it also enables individuals to engage reflexively with their configurations. By recalibrating their dispositions to maintain alignment with shifting relational logics, individuals contribute to the adaptive resilience of social configurations, reinforcing their structures while facilitating incremental transformation (Cook et al., 2012).

##### 4.5.6.2 Doxa: configurational beliefs and naturalized norms

Doxa, as reinterpreted within CFA, refers to the naturalized norms and values that underpin the implicit logics of configurations. These beliefs are internalized through configurational habitus, shaping individuals’ perceptions and practices as unquestioned truths that sustain the coherence and legitimacy of relational structures (Bourdieu, 1990; Swartz, 1997). For instance, in the academic field, the belief in the objectivity and meritocracy of peer-reviewed publishing functions as a configurational doxa. This belief anchors the practices of academics, guiding their actions in ways that align with the field’s hierarchical logics. However, these naturalized norms also reinforce existing power dynamics, privileging those who possess the resources and cultural capital to navigate the field effectively (Davey, 2012; Hunter, 2004). Configurational doxa is both a stabilizing and constraining force. While it ensures the continuity of relational structures, it also embeds inequalities within configurations, making them appear intuitive and inevitable. Yet, as individuals engage reflexively with their habitus, they may question or challenge these naturalized norms, creating spaces for resistance and reconfiguration. This tension between stability and critique underscores the dual role of doxa in sustaining and transforming social configurations.

##### 4.5.6.3 Crisis: reflexive recalibration and structural reconfiguration

Crisis represents a rupture in the alignment between configurational habitus and the relational structures of a field. These moments of disruption expose the contingent nature of social configurations, prompting reflexive recalibration as individuals adapt to new relational dynamics. For example, technological innovations or sociopolitical upheavals may destabilize established configurations, creating a mismatch between internalized dispositions and emerging field logics. Workers in traditional industries may need to acquire new skills or shift to entirely different configurations, while policymakers must navigate the relational complexities of restructured governance systems (Beck, 2000; Wacquant, 2016). In CFA, crisis is not merely a moment of disruption but a site of transformative potential. By revealing the relational and contingent foundations of social configurations, crisis enables individuals to reassess and reconfigure their practices, fostering innovation and adaptability (Karner, 2005; Kerr and Robinson, 2011). This reflexive recalibration highlights the resilience and flexibility of configurational habitus, demonstrating its capacity to sustain coherence while facilitating relational transformation.

## 5 Conclusion

The development of Configurational Field Analysis (CFA) as presented in this paper represents both a theoretical and methodological evolution, addressing the limitations inherent in Bourdieu’s Field Theory and the idea of Global Field and, more broadly, in traditional approaches to social analysis within global contexts. By embracing the fluidity, indeterminacy, and contextual contingency of social configurations, CFA offers a novel framework that moves beyond the structural determinism and Eurocentrism that have historically constrained field theory. This reconfiguration does not merely modify existing theoretical structures; rather, it reconstructs the foundational concepts of field, capital, and habitus, embedding them within a dynamic and relational understanding of global phenomena. Thus, it can be argued that, unlike the adjustments made to the theory of the global field to accommodate the dynamics of an interconnected world, reinterpreting this theory through the lens of social configurations could create a more effective and flexible analytical tool. This approach goes beyond merely shifting scope or scale or refining aspects of the social field theory; it provides a foundation for analyzing a world where cosmopolitanization, grounded in a distinct ontology, introduces a new logic in the construction of social phenomena.

The CFA reconceptualizes social spaces as continuously evolving and contextually embedded fields where power, capital, and influence are constantly renegotiated. By recognizing the historical and situational contingency of social configurations, this framework challenges the static and universal assumptions that have often underpinned global theories. Through this lens, global social phenomena are understood not as fixed entities governed by deterministic logics but as fluid and relational processes shaped by the interactions of actors within specific historical, cultural, and political contexts. This approach allows for a more nuanced and adaptable analysis, capable of capturing the complexities and uncertainties inherent in contemporary global dynamics. The implications of CFA extend beyond the theoretical realm, offering practical insights for empirical research. CFA provides a robust analytical tool sensitive to the multiplicity of factors influencing global phenomena, allowing researchers to dissect the relational dynamics at play across different scales of social life. This methodological flexibility enables the study of global interactions in a way that is both contextually grounded and theoretically rigorous, bridging the gap between abstract theory and concrete empirical observation. Furthermore, by integrating the concept of social configurations with Bourdieu’s field theory, CFA offers a comprehensive framework particularly well-suited to analyzing the transnational flows of capital, power, and cultural influence that characterize the contemporary global landscape.

In advancing this framework, this paper has not only addressed critiques of Global Field Theory but has also laid the groundwork for future research. The introduction of social configurations as dynamic, contingent constructs opens new avenues for investigating how global fields are continuously reconstituted through historical processes and relational interactions. This rethinking of fields calls for further exploration into the intersections between local and global forces, and how these interactions shape both the stability and transformation of global structures.

Future research should extend the application of CFA to diverse empirical contexts, testing its utility across various domains such as transnational politics, global economics, and cultural production. Additionally, there remains a need to refine the theoretical distinctions within CFA, particularly regarding how different forms of capital interact and transform within specific configurations. By continuing to develop and apply this framework, scholars can gain deeper insights into the evolving nature of global phenomena and the complex forces that drive them.

## Data Availability

The original contributions presented in the study are included in the article/supplementary material, further inquiries can be directed to the corresponding author.
